# Data on location and retail price of a standard food basket in supermarkets across New York City

**DOI:** 10.1016/j.dib.2023.109222

**Published:** 2023-05-09

**Authors:** Aldo Crossa, Eli Cooperman, Breanna James, Stephen Ma, María Baquero

**Affiliations:** Bureau of Epidemiology Services, New York City Department of Health and Mental Hygiene, USA

**Keywords:** Food pricing, Food access, Standard food basket

## Abstract

Previous work has suggested that the price of food sold at supermarkets may vary according to the socioeconomic characteristics of a neighborhood. Given the importance of food prices in securing access to food, understanding how food prices vary across neighborhoods is crucial to assessing affordability. To study food pricing in New York City (NYC) a defined standard food basket (SFB) was collected in supermarkets across NYC neighborhoods. A dataset was created that includes pricing data collected in-person for ten pre-determined food items from 163 supermarkets across 71 of the 181 NYC neighborhoods during March through August of 2019. Included in these data are raw and processed pricing data files that illustrate the complexity of standardizing pricing across items. An additional dataset includes neighborhood-level variables of selected socioeconomic and demographic characteristics from the 2014–2018 American Community Survey that is publicly available via the Census API. The pricing data and the data on neighborhood-level characteristics were merged. Basic statistical measures suggest some distributional differences in the price of a SFB by socioeconomic differences between neighborhoods. This database can be used to describe spatial patterns in food pricing in a dense urban setting, while exploring pricing inequities across neighborhoods. In addition, by working with these data, researchers, policy analysts and educators will gain an understanding of the methodologies used to generate pricing data for an SFB.

**Specifications Table**

**Table:** 

Subject	Health Economics
Specific subject area	Distribution of food prices in supermarkets across New York City neighborhoods
Type of data	Dataset Data dictionary Table Figures
How the data were acquired	New York City Department of Health and Mental Hygiene (DOHMH) personnel conducted in-person visits to 163 supermarkets across New York City between March and August 2019 to record the price of 10 food items that make up a Standard Food Basket (SFB). The data were collected via the mobile application Survey123. The raw data were processed using R to generate an analytic dataset. ZCTA-level data were sourced from the publicly available 2014–2018 American Community Survey data.
Data format	Raw Analyzed
Description of data collection	We defined a reduced SFB with 10 perishable items based on reports of items purchased by Supplemental Nutrition Assistance Program recipients[1]. A preferred version of each food item, including selling unit (pound, container, etc.) and type was determined a priori for data collection. Price of each food item was recorded from 163 supermarkets across 71 of the 181 populated ZIP code tabulation areas in New York City.
Data source location	• Institution: Various supermarkets • City/Town/Region: New York City, New York • Country: United States of America Data are accessible in a GitHub repository (see Data accessibility section).
Data accessibility	Data are accessible in the following GitHub repository: Repository name: **food-pricing-survey-nyc-2019** Data identification number: **DOI:**10.5281/zenodo.7896745 Direct URL to data: https://github.com/nychealth/food-pricing-survey-nyc-2019/

## Value of the Data

• This is a primary dataset of retail food prices in 71 of 181 ZIP code tabulation areas across New York City, collected by a municipal health department using low-cost methods. In addition to comparing standardized prices across New York City neighborhoods, it includes other information that may influence the price (e.g., whether an item is organic).
• Researchers interested in market pricing, food environments, and food insecurity in metropolitan urban settings may gain knowledge to conduct similar epidemiological studies of social determinants of health.
• In addition, by working with these data, researchers, policy analysts and educators will gain an increased understanding of generating pricing data for a standard food basket.

## 1 Data Description

### 1.1 Calculating Neighborhood-Level Indicators

In public health research, a neighborhood can be characterized via sociodemographic measures of the population and indicators of economic wealth. Some of those measures are often used by the NYC DOHMH to highlight inequities resulting in persistent structural and institutional racism and unjust policies[2]. In this analysis, we used data from 2014 to 2018 ACS extracted via the Census API to generate the measures and indicators of interest [3].

Each of the ZIP code tabulation areas (ZCTA) were categorized according to demographics (race/ethnicity, US-born population), socioeconomic characteristics (SNAP participation, poverty, educational attainment, employment and linguistic isolation), home ownership, and the estimated Gini coefficient (a summary measure of income inequality) at the ZCTA-level. A description of the selected neighborhood-level variables is presented in Table 3.

### 1.2 File 1: Raw Pricing Data

Raw data for each food item and the location of stores visited are included in the dataset in File 1. (Raw_Pricing_data_final.csv). The dataset includes information on whether a preferred or alternative version (e.g., presentation, packaging) of each food item was identified in the store, item brand name, whether the item was on sale, as well as a flag indicating whether the item was organic [4,5]. A data dictionary for all variables in the raw dataset is included in Supplementary file 4.

### 1.3 File 2: Processed Pricing Data

Once the data collection was completed in the stores by personnel from the NYC Department of Health and Mental Hygiene (DOHMH), the data were downloaded and processed to make an analytic dataset. The data processing included consolidating the price of preferred and alternate options into a single variable and imputing missing values. **File 2.** (Cleaned_Pricing_data_imputed_final.csv) contains consolidated and imputed variables for each food items in addition to the cost of the SFB made from the ten pre-selected items.

**Fig. 1:**
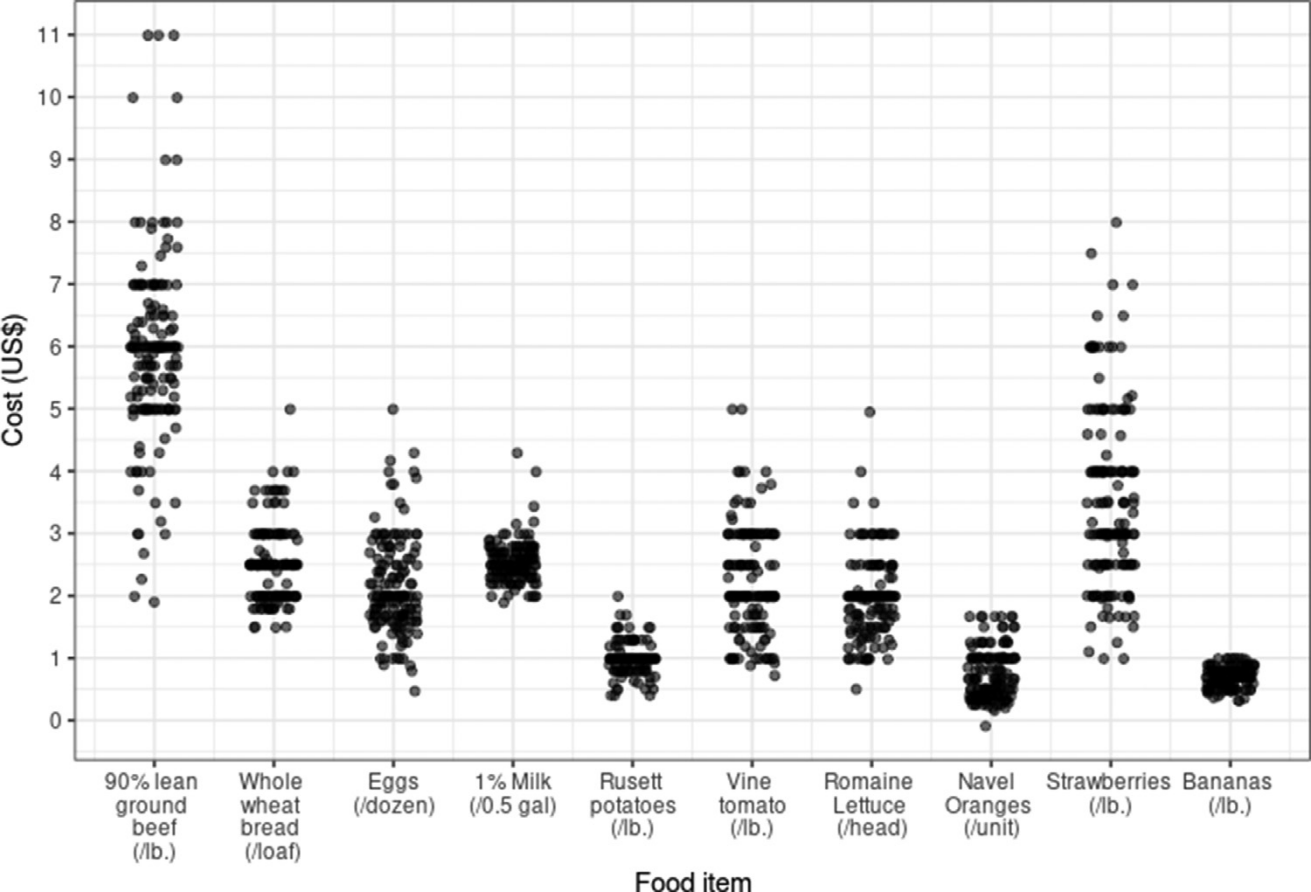
Distribution of normalized pricing for each of the ten items included in the SFB. Each point represents the price of that item at a store and includes imputed values.

**Fig. 2:**
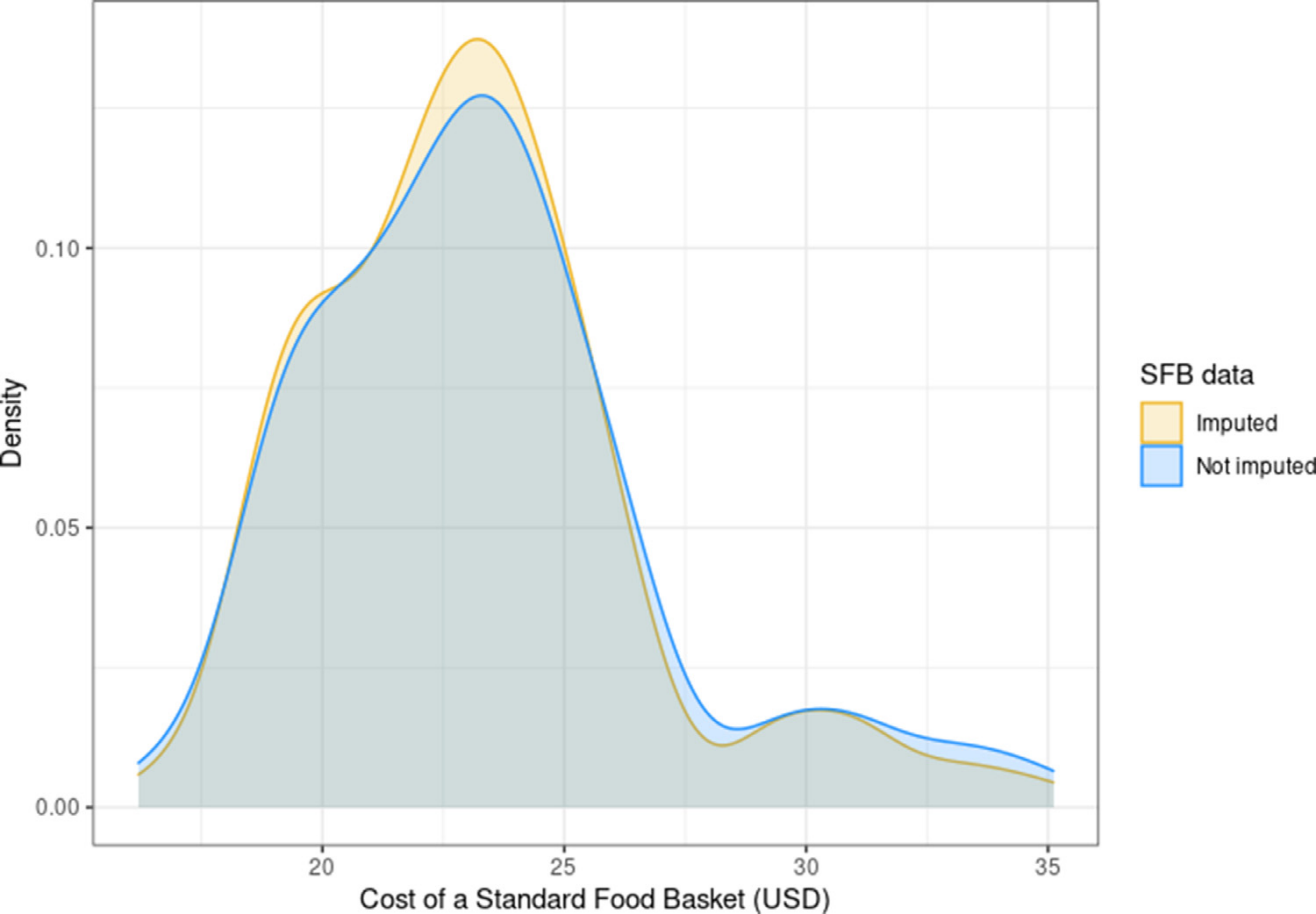
Distribution of the standard food basket in supermarkets across New York City in 2019, with all not-imputed values and imputed values.

Fig. 1, Fig. 2: Variability of food pricing and distribution of SFB price.

Fig. 1 shows the variability in the pricing of each of the food items in the SFB.

Using the data collected from the 163 supermarkets visited, we explored the price distribution of the food items in the SFB. We plotted the distribution of the SFB first excluding any cases with missing values and then with imputed SFB values included where necessary. The density distribution of the SFB is illustrated in Fig. 2. The calculated SFB for the overall dataset was $22.81 (range = $16.20 – $35.11, IQR = $3.94), with positive skewness (SK=1.00) indicating a non-symmetric distribution with a larger tail towards the higher priced end of the distribution (see Fig. 2). The calculated kurtosis (KR=4.46) suggests a more flattened distribution.

### 1.4 File 3: Neighborhood Level Characteristics

**File 3** (Neighborhood indicators_final.csv) provides calculated values for the indicators listed in Table 1 for each of the 181 ZIP code tabulation areas within New York City. In addition to the estimated value for each indicator (and its associated error), it also includes each indicator categorized according to the quartiles (25th, 50th, 75th, and 100th percentile) based on the values.

**Table 1:** Description of variables from 2014 to 2018 American Community Survey pulled via the Census API to calculate from the ZCTA-level indicators.

Characteristic	Description of indicator (API variables)
Housing ownership and rent	
Home Ownership	
	Percent of households that are owner occupied
	B25003_002E/B25001_001E
Rent Burden	
	Percent of renter-occupied households where the rent is 30% or more of the total household income.
	(B25106_028E+B25106_032E+B25106_036E+B25106_040E+B25106_044E)/ B25106_024E
House Value^a^	
	Median value of the household
	B25077_001E

Socioeconomic characteristics	

SNAP Enrollment	
	Percent of all households that received Food Stamps
	B22001_002E/B22001_001E
Poverty	
(<100% FPL^b^)	
	Percent of the population living under the federal poverty line
	(C17002_002E+C17002_003E)/C17002_001E
Employed	Percent of the population over the age of 16 that were employed
	B23025_004E/B23025_001E
Didn't Complete High School	
	Percent of the population over the age of 24 that didn't complete high school
	B06009_002E/B06009_001E
Not English speaking	
	Percent of the population aged 14 or older that report speaking English “not well” or “not at all”.
	(B16005_007E+B16005_008E+B16005_012E+B16005_013E+B16005_017E+B16005_018E+B16005_022E+B16005_023E+B16005_029E+B16005_030E+B16005_034E+B16005_035E+B16005_039E+B16005_040E+B16005_044E+B16005_045E)/B16005_001E

Race/Ethnicity	

% Black/	
Black American	Percent of the population that identifies as Black or African American.
	B02009_001E/B02001_001E
% Hispanic/Latino	Percent of the population that identifies as Hispanic or Latino/a.
	B03001_003E/ B03001_001E
% Asian	Percent of the population that identifies as Asian or Pacific Islander.
	B02011_001E/B02001_001E
Other	Percent of the population that identifies as Other.
	B02013_001E/B02001_001E

Health Insurance	

No Health Insurance	Percent of the population that reports not having insurance
	(B27010_017E+B27010_033E+B27010_050E+B27010_066E)/ (B27010_002E+B27010_018E+B27010_034E+B27010_051E)

Neighborhood inequality	

Gini Index	Estimated ZCTA-level GINI-coefficient
	B19083_001E

a House Value expressed in US dollars, *K*=$1000.

b Federal Poverty Line for 2018 as defined by the U.S. Department of Health and Human Services.

Table 2: Quartile ranges of neighborhood characteristics for selected indicators, New York City 2013–2018.

**Table 2:** Distribution of the value of selected indicators for the 181 ZIP code tabulation areas in New York City 2013–2018.

Characteristic	Q1 Range (%)	Q2 Range (%)	Q3 Range (%)	Q4 Range (%)
Housing ownership and rent				
% Owner occupied homes	2.9%−16.2%	16.2%−30.1%	31.4%−48.8%	49%−87.4%
% Rent burdened^a^	22.6%−42.4%	42.5%−49.3%	49.4%−54.7%	54.8%−70.4%
Median house value^b^	165K-465K	469K-583K	589K-814K	835K-2000K+

Socioeconomic characteristics				
% Enrolled in SNAP^c^	0.2%−6.8%	7%−14.3%	14.3%−24.6%	24.7%−53.2%
% Households with income <100% FPL^d^	2.2%−8.7%	9.1%−13.1%	13.1%−20.8%	20.8%−47.9%
% Employed	41.3%−55.6%	55.6%−60%	60.1%−64.7%	65.3%−88.3%
% That didn't complete HS	0.6%−9.2%	9.7%−15%	15%−21.9%	21.9%−46.4%
% Not an English speaker^e^	0.2%−3.6%	3.7%−7.8%	7.9%−14.9%	14.9%−41.5%

Race/Ethnicity				
% Black/ Black American	0%−4.6%	4.7%−10.7%	11%−36.5%	36.5%−94.2%
% Hispanic/Latino	3.2%−11.5%	11.5%−18.4%	18.8%−37.5%	37.5%−76.8%
% Asian	0.5%−5%	5%−11.3%	11.5%−24.4%	24.9%−73.6%
Health Insurance				
% with no health insurance	0%−4.3%	4.3%−6.6%	6.6%−8.2%	8.3%−23.4%
Neighborhood inequality				
Gini Index^f^	0.376–0.435	0.435–0.474	0.474–0.522	0.523–0.628

a Among those who report renting their home, individuals are rent burdened if the household income is the amount paid in rent is >30% of the household income.

b House Value expressed in thousands of US dollars.

c Supplemental Nutrition Assistance Program.

d Among individuals over age 14, percent that report speaking English “not well” or “Not at all”.

e Federal Poverty Line for 2018 as defined by the U.S. Department of Health and Human Services.

f Gini Index is a summary measure of income inequality.

The range of values by quartile in each of the categories is presented in Table 2.

**Fig. 3:**
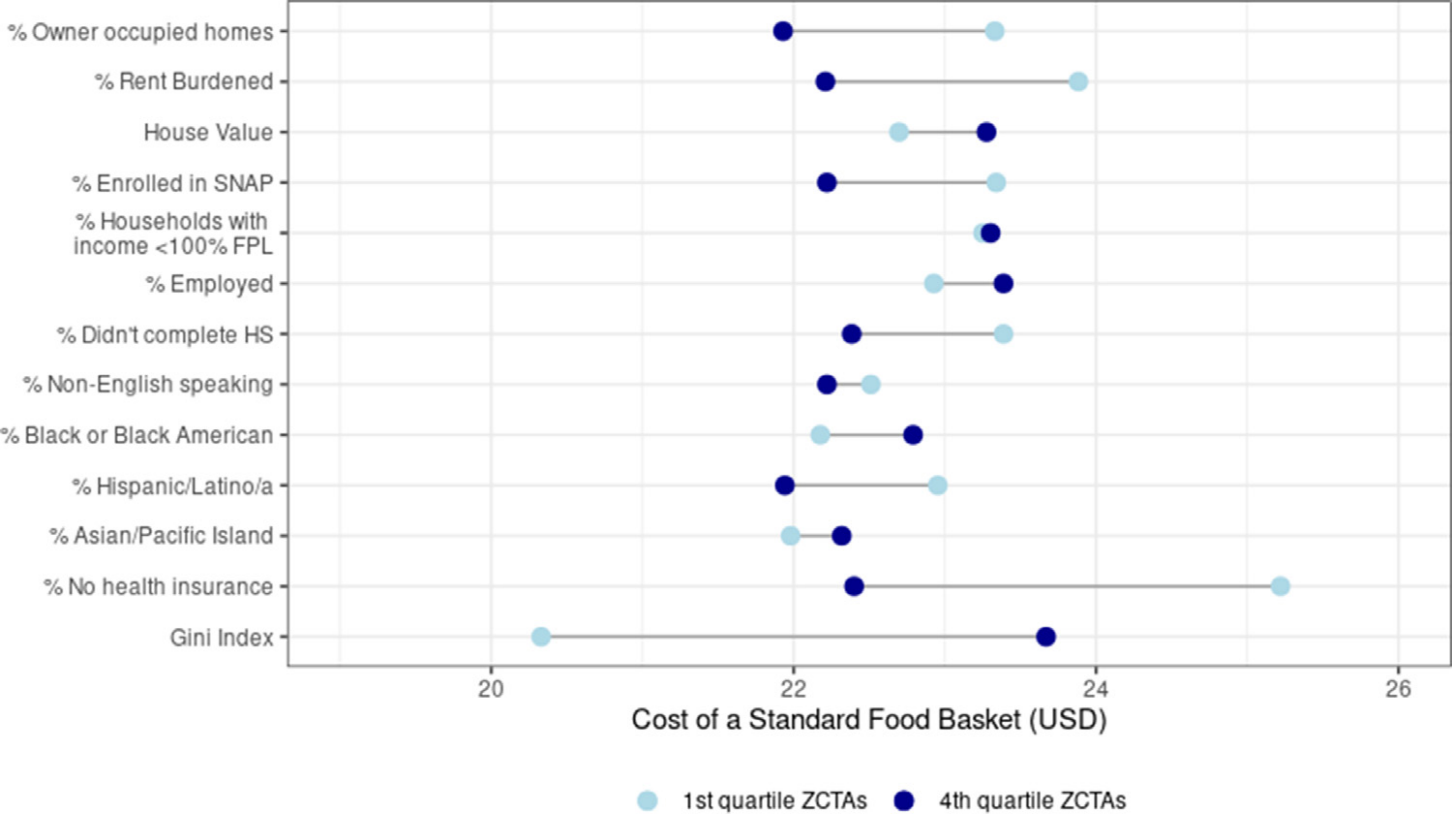
Comparison of the median SFB between ZIP code tabulation areas in the highest and lowest quartile for each of the neighborhood level estimates. ^1^Among those who report renting their home, individuals are rent burdened if the household income is the amount paid in rent is >30% of the household income; ^2^House Value expressed in US dollars, *K*=$1000; ^3^Supplemental Nutrition Assistance Program; ^4^Among individuals over age 14, percent that report speaking English “not well” or “Not at all”; ^5^ Federal Poverty Line for 2018 as defined by the U.S. Department of Health and Human Services; ^6^ Gini Index is a summary measure of income inequality.

**Fig. 4:**
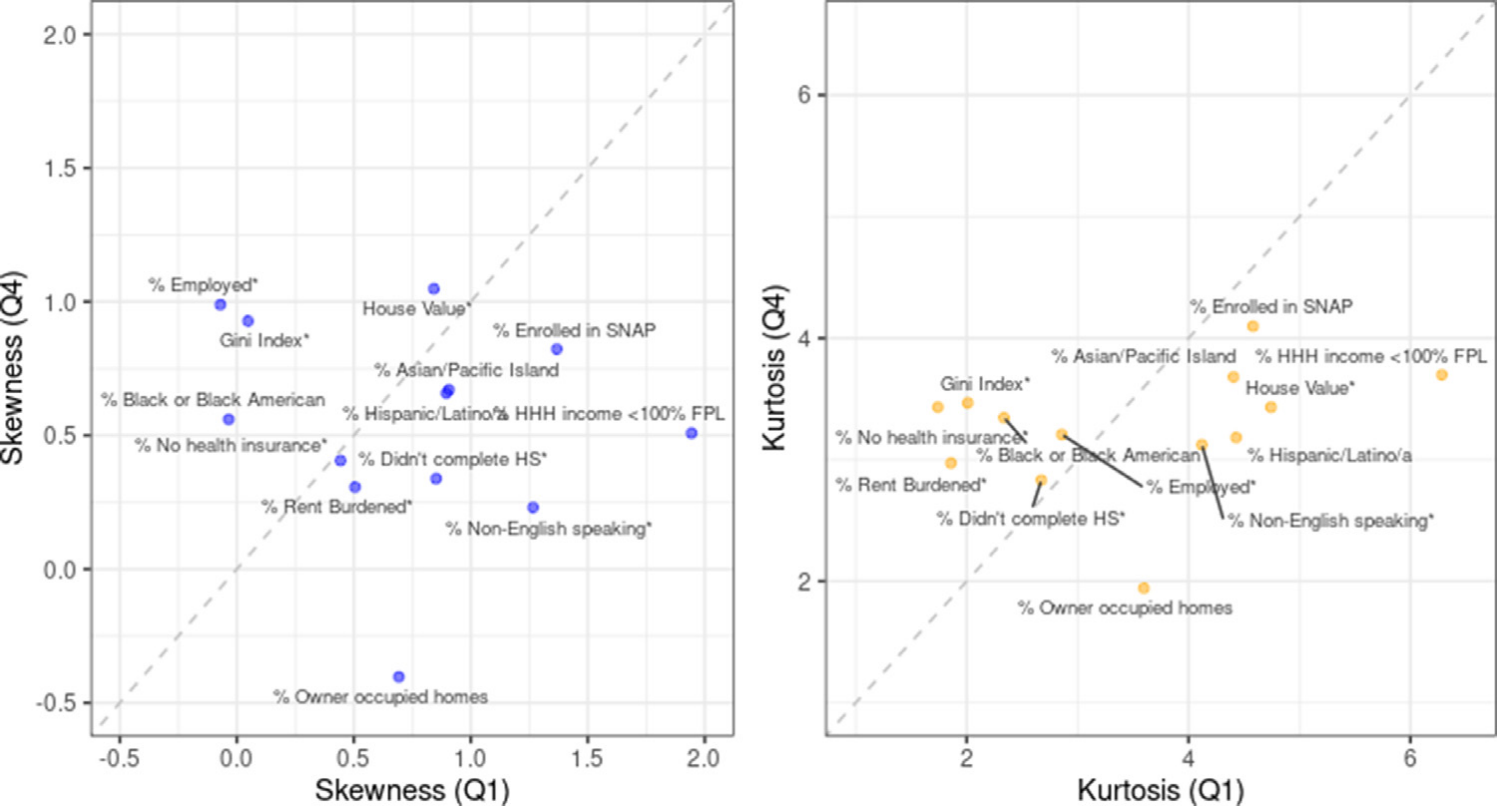
Visualization of the calculated values for skewness and kurtosis for the distribution of SFB among ZIP code tabulation areas (ZCTA) in the top and bottom quartiles (Q1 and Q4) by each ZCTA level indicator. New York City 2019.

Fig. 3, Fig. 4: Distribution of SFB price by neighborhood characteristics.

The median price of the SFB varied according to the socioeconomic characteristics of a ZCTA (see Fig. 3). The largest difference in median SFB price was between ZCTA in the lowest and highest quartiles of the Gini index ($20.33 vs $23.67 respectively) though the measures of skewness and kurtosis were similar (see Fig. 4). ZCTAs with the highest percentage of uninsured residents had a lower median SFB price ($22.40) and compared to ZCTAs with a lower percent of uninsured residents (median = $25.22). While ZCTAs in the highest and lowest quartiles by income had a similar median price of the SFB ($23.30 vs. $23.25 respectively), the values were for skewness (1.94 vs 0.51 respectively) and kurtosis (6.28 vs. 3.70 respectively) were different.

## 2 Experimental Design, Materials and Methods

### 2.1 Defining a Standard Food Basket (SFB)

Prior to data collection, we defined an SFB of 10 perishable food items that included a variety of food types (see Table 3). The specific items chosen for the SFB included elements from USDA's My Plate guidelines and were based on the report Foods Typically Purchased by Supplemental Nutrition Assistance Program (SNAP) Households [1]. Our SFB was also informed by previous work reported by the Hunter College NYC Food Policy Center [5].

To reduce the variability in type and quantity of each item in the SFB, we defined item-level parameters to guide the data collection once in the store. First, we defined a “preferred item presentation” that the data collector should identify first. This preferred item presentation was determined based on amount per selling unit (e.g., pound or gallon), variety (e.g., “Vine tomatoes”), and other characteristics (e.g., leanness). If the exact preferred item presentation was not identified in the store, then an alternative item was chosen, based on considerations as described in Table 3. For four items (eggs, bananas, whole wheat bread and strawberries) no alternative item was defined based on in-store observations done prior to data collection. Finally, having identified the item (preferred presentation or alternative item), the variety sold at the lowest price was recorded in the database.

**Table 3:** Preferred item presentation and considerations for selecting an alternative item for the 10 items used in the SFB. The comments include some assumptions during the data processing steps to normalize the price of alternative options.

Item	Preferred item presentation	Considerations for selecting alternative item	Comment on normalizing the price of the alternative item.
Beef	1 lb.; 90% lean	Closest leanness, weight	Calculate price per lb.
Eggs	12 Large; Brown	*No alternative*	*N/A*
Milk	½ gallon 1%	Closest in fat content, size	Calculate price per ½ gallon.
Oranges	1 unit; navel oranges	Closest in size	Assumed 3.2 oranges per pound
Tomatoes	1 lb.; vine tomatoes	Closest in size (e.g., no cherry tomatoes)	Assumed 2.5 tomatoes per lb.
Potatoes	1 lb.; Russet variety	Smallest bag size available; avoid using the price of packages with 1 or 2 potatoes.	Assumed 2.5 potatoes per lb.
Bananas	1 lb.	*No alternative*	*N/A*
Bread	1 sliced loaf; whole wheat	*No alternative*	*N/A*
Strawberries	1 lb. container	*No alternative*	*N/A*
Lettuce	1 head of lettuce; Romaine variety	Iceberg by the head	Calculate price per head of lettuce. 1 head of romaine lettuce weighs 1.375lbs.

### 2.2 Collecting Price Data

The prices of individual items in the SFB were collected using a tool adapted from the Nutritional Environment Measures Survey in stores (NEMS-S), a validated tool designed to collect data on price and quality in retail food stores [6]. The adapted NEMS tool was implemented on the digital platform Survey123 (Environmental Systems Research Institute, ESRI) so that data collection could be done on mobile devices. Mobile data collection was crucial to the viability of a data collection project with limited resources. In addition to price data, other information collected included item brand name, whether the item was on sale and whether it was organic, given its influence on price [2,3].

### 2.3 Sampling Supermarkets

Previous work has suggested that there may be some differences in the price of food according to characteristics of a neighborhood [7], [8], [9]. To capture as much of the variability across New York City neighborhoods as possible, we aimed to sample at least one supermarket from each of the 55 community districts in New York City. With the resources available, we reached 52 of the 55 PUMA neighborhoods and covered 71 of the 181 populated ZCTAs in NYC. Although individual supermarkets were chosen through purposeful sampling based on accessibility by public transport, we prioritized gentrifying neighborhoods (as defined by the NYU Furman Center[10]) and supermarkets that were either corporate chains or part of “voluntary associations” that are independently owned but uniformly branded (e.g. “Key Food”).

Importantly, the sampling strategy introduces potential biases to how representativeness this data is of the general food landscape in NYC. Supermarkets that are accessible via public transit may not be representative of the universe of supermarkets in NYC. There are densely populated regions of NYC that are at the margins of the public transportation network, with reduced access to the subway and rail systems. As a result of historically racist and unjust transit policy, people that live in these regions are predominantly from low-income households, are people of color or immigrants[11]. While we made a concerted effort to include supermarkets accessible only via bus (the bus network is accessible to 99% of the NYC population [12]), users of this data should consider this potential source of bias in the dataset.

### 2.4 Calculating the SFB

Once pricing data were collected at supermarkets, we took steps to process the data in order to calculate the SFB for each supermarket.

We first normalized the item-level price data for alternative items collected in accordance with the comments on normalizing described in Table 3. The normalization of the price of the alternate items was intended to make the price of the alternative item comparable the pricing of the preferred item. After completing the normalization, we consolidated the variables of preferred and alternative item prices into a new, single variable in the database (resulting in 10 price variables, one for each item). In addition, for each of the 10 normalized price variables, we generated a version where any missing values where imputed. Missing price data for each item was imputed via multiple imputation, using the observed neighborhood and item price to generate imputed values. All imputation was done using the mice package in R. Finally, the SFB for a supermarket was calculated by summing the consolidated and imputed cost of all 10 items.

### 2.5 Merging Price Data to Neighborhood-Level Indicators

We identified the corresponding public-use microdata areas for each supermarket visited using the address data and used public-use microdata areas as a linking variable to merge it with the neighborhood-level characteristics data. This data merge allowed us to obtain SFB price estimates at the neighborhood level and by neighborhood characteristics.

### 2.6 Calculating Distributional Properties of SFB by Neighborhood Characteristics

We calculated median, skewness and kurtosis to characterize the distribution of the calculated SFB. These distributional measures were calculated for the overall sample of 163 supermarkets visited. We then calculated the same distributional measures for neighborhoods as grouped according to the selected neighborhood level indicators. Finally, we visualized the differences in the calculated median, skewness, and kurtosis for the SFB for neighborhoods in the highest versus the lowest quartiles of the distributions for each of the selected characteristics.

## Ethics Statements

The authors have no conflicts of interest to report.

## CRediT authorship contribution statement

**Aldo Crossa:** Conceptualization, Methodology, Software, Formal analysis, Visualization, Writing – original draft, Writing – review & editing. **Eli Cooperman:** Conceptualization, Methodology, Data curation, Writing – original draft, Writing – review & editing. **Breanna James:** Methodology, Data curation, Writing – original draft, Writing – review & editing. **Stephen Ma:** Formal analysis, Visualization, Writing – review & editing. **María Baquero:** Conceptualization, Methodology, Visualization, Writing – review & editing, Supervision.

## Declaration of Competing Interest

The authors declare that they have no known competing financial interests or personal relationships that could have appeared to influence the work reported in this paper.

## Data Availability

NYC food pricing (Original data) (GitHub).
